# Changing genomic epidemiology of COVID-19 in long-term care facilities during the 2020–2022 pandemic, Washington State

**DOI:** 10.1186/s12889-023-17461-2

**Published:** 2024-01-15

**Authors:** Hanna N. Oltean, Allison Black, Stephanie M. Lunn, Nailah Smith, Allison Templeton, Elyse Bevers, Lynae Kibiger, Melissa Sixberry, Josina B. Bickel, James P. Hughes, Scott Lindquist, Janet G. Baseman, Trevor Bedford

**Affiliations:** 1 Department of Health, Washington State, 1610 NE 150th St, Shoreline, Washington 98155 USA; 2 University of Washington, 1410 NE Campus Parkway, Seattle, Washington 98195 USA; 3 Yakima Health District, 1210 Ahtanum Ridge Dr, Union Gap, Washington 98903 USA; 4 Fred Hutchinson Cancer Research Center, 1100 Fairview Ave N, Seattle, Washington 98109 USA

**Keywords:** Epidemiology, Surveillance, SARS-CoV-2, Genomics, Healthcare-associated Infections, Public health

## Abstract

**Background:**

Long-term care facilities (LTCFs) are vulnerable to disease outbreaks. Here, we jointly analyze SARS-CoV-2 genomic and paired epidemiologic data from LTCFs and surrounding communities in Washington state (WA) to assess transmission patterns during 2020–2022, in a setting of changing policy. We describe sequencing efforts and genomic epidemiologic findings across LTCFs and perform in-depth analysis in a single county.

**Methods:**

We assessed genomic data representativeness, built phylogenetic trees, and conducted discrete trait analysis to estimate introduction sizes over time, and explored selected outbreaks to further characterize transmission events.

**Results:**

We found that transmission dynamics among cases associated with LTCFs in WA changed over the course of the COVID-19 pandemic, with variable introduction rates into LTCFs, but decreasing amplification within LTCFs. SARS-CoV-2 lineages circulating in LTCFs were similar to those circulating in communities at the same time. Transmission between staff and residents was bi-directional.

**Conclusions:**

Understanding transmission dynamics within and between LTCFs using genomic epidemiology on a broad scale can assist in targeting policies and prevention efforts. Tracking facility-level outbreaks can help differentiate intra-facility outbreaks from high community transmission with repeated introduction events. Based on our study findings, methods for routine tree building and overlay of epidemiologic data for hypothesis generation by public health practitioners are recommended. Discrete trait analysis added valuable insight and can be considered when representative sequencing is performed. Cluster detection tools, especially those that rely on distance thresholds, may be of more limited use given current data capture and timeliness. Importantly, we noted a decrease in data capture from LTCFs over time. Depending on goals for use of genomic data, sentinel surveillance should be increased or targeted surveillance implemented to ensure available data for analysis.

**Supplementary Information:**

The online version contains supplementary material available at 10.1186/s12889-023-17461-2.

## Introduction

The COVID-19 pandemic disproportionately impacted residents of long-term care facilities (LTCFs), who have suffered higher mortality rates than the general population; in Washington State (WA), LTCF-associated cases represent 3% of cases, but 30% of deaths due to SARS-CoV-2 [1]. This impact materialized in WA and across the US despite early recognition of LTCFs as high-risk settings due to residents’ advanced age, chronic underlying health conditions, congregate living, asymptomatic transmission, and movement of healthcare personnel [2–4].

Based on these concerns, Centers for Disease Control and Prevention (CDC) developed recommendations over the course of the pandemic for infection prevention and control (IPC) in LTCFs, including training, use of personal protective equipment (PPE) and hygiene measures, visitor restrictions, resident distancing and cohorting, environmental cleaning and disinfection, testing and reporting to public health jurisdictions, and provision of staff sick leave [5].Similarly, WA’s governor, secretary of health, and Department of Health (DOH) developed and instituted regulations and guidance governing prevention efforts [6, 7]. Centers for Medicare and Medicaid Services (CMS) outlined rules for testing staff and residents of LTCFs [8]. Changes in these rules, regulations, and guidance over time are expected to have impacted transmission dynamics in LTCF settings.

One key tool for understanding transmission dynamics in-place is pathogen genomic sequencing and analysis, particularly phylogeographic analysis. Understanding sampling methodology is important for describing potential bias in this type of analysis [9–11]. Systems for sequencing SARS-CoV-2 specimens have changed over time. Prior to March 2021, sampling for sequencing from WA residents was convenience- or research-based. In March 2021, a sentinel surveillance system was implemented in WA to support representative sampling [9]. The population of WA LTCF-associated cases with genomic data available is as-yet undescribed. Additionally, the utility of the existing surveillance system for adding insight and actionable data for public health practice has not been completely explored.

Multiple examples of genomic epidemiology studies of single outbreaks or facilities exist in the literature, including from WA. A previous study documented the utility of targeted genomic surveillance during two SARS-CoV-2 outbreaks in LTCFs in WA [12]. Likewise, a study of a single LTCF-associated outbreak in WA early in the pandemic utilized genomic epidemiology to understand phylogenetic clustering of cases within the facility [13]. Fewer studies have leveraged pathogen genomic data to describe how transmission dynamics changed over the pandemic or describe the impact of sequence data availability on public health action. A review article assessing published genomic epidemiologic investigations during 2020 documented the value of this type of analysis for identifying independent clusters of infections but found that large-scale sequencing of outbreaks added limited value after sequencing initial cases, focusing on individual outbreak- or facility-level studies [14]. An analysis of all care-home linked cases in the east of England used genomic epidemiology to explore large-scale transmission dynamics in nearly 300 facilities; however, this analysis was limited to a 3-month study period [15].

Here, we aim to assess the utility of genomic data produced for LTCF-associated cases to add information for public health action over the course of the SARS-CoV-2 pandemic, from 2020–2022. We pair patient-level epidemiological and pathogen genomic data to understand variations in transmission patterns over time. Specifically, we address the following questions of public health concern: is available genomic data obtained from LTCF-associated cases representative of all LTCF-associated cases? Do temporal changes in guidance or policy apparently impact intra-facility transmission patterns? Given available data, which genomic-epidemiologic methods are most applicable for ongoing or routine data analysis? And finally, what changes are needed to ensure the ongoing use of genomic data to explore transmission in LTCF settings?

## Methods

### Data collection and cleaning

All confirmed COVID-19 cases reported among WA residents in the Washington Disease Reporting System (WDRS) as of December 19, 2022 were included, including reinfection cases [16]. Sequences uploaded to the GISAID EpiCoV database indicating WA in their geographic tag were linked to these cases using laboratory accession numbers or patient demographics [17]. For cases with multiple specimens sequenced, only the first specimen was used for analysis. Long-term care facilities were defined as: nursing homes, assisted living facilities, adult family homes, enhanced services facilities, and intermediate care facilities for individuals with intellectual disabilities. Cases in WDRS are categorized as LTCF-associated if association with a facility is noted in case interview, medical record, facility line list, address or telephone match to the facility or another measure indicated by the Local Health Jurisdiction. LTCF-associated cases therefore include residents, employees, and visitors if association is noted.

Enhanced data obtained on October 24, 2022 from Yakima Health District tracking additional details related to LTCF cases and outbreaks were linked to WDRS and GISAID data using name and date of birth and conducting probabilistic matching with manual review.

### Representativeness analysis

All epidemiological data analysis was performed in R version 4.2.2 [18]. Representativeness of LTCF-associated cases with sequencing performed was assessed by comparing to all LTCF-associated cases on: sex, age, race, ethnicity, language, outbreak association, symptom status, hospitalization, death, and facility type. Sampling for sequencing over time in the full population and in LTCFs was graphed.

### Definition of study time-periods

Information available from the WA Governor’s News Release Archive and WA DOH records was used to construct a timeline of key modifications to rules, regulations, or guidance for LTCFs. This timeline was used to divide the study period into six segments of approximately similar lengths, marked by key policy changes (Table 1). Events that impacted movement or visitation and sample selection for sequencing were prioritized in defining study time-periods.

**Table 1:** Dates and key events defining each study time-period, 1–6

*Study Period*	*Event Date*	*Event Description*
*1 (Jan 20,2020-Mar 9, 2020)*	Jan 20, 2020	First COVID-19 case confirmed in WA
*2 (Mar 10, 2020-Aug 11, 2020)*	Mar 10, 2020	Governor issues rules to restrict LTCF visitation, require visitor screening, and require isolation of residents testing positive for SARS-CoV-2
	Mar 23, 2020	Stay home, stay healthy order
	Jun 26, 2020	First statewide masking order takes effect
*3 (Aug 12, 2020-Mar 9, 2021)*	Aug 12, 2020	Updated LTCF visitation guidance allows for increased visitation
	Aug 25, 2020	Centers for Medicare & Medicaid Services (CMS) releases testing requirements for LTCF staff and residents
	Nov 15, 2020	LTCF visitation restrictions re-instituted
	Dec 20, 2020	LTCF vaccination campaign begins
	Mar 1, 2021	Sentinel sampling for genomic sequencing initiated
*4 (Mar 10, 2021-Aug 22, 2021)*	Mar 10, 2021	Masking and visitation restrictions lifted for fully vaccinated
	Mar 17, 2021	Second phase of vaccine roll-out begins
	Mar 19, 2021	Indoor LTCF visitation allowed if visitor or resident is fully vaccinated
	Apr 1, 2021	LTCF vaccination campaign complete
	Apr 15, 2021	Vaccines available for everyone aged 16 +
	Jul 1, 2021	Implemented the 10/70 rule for visitation in LTCFs: indoor visitation restricted only for unvaccinated residents in facilities located in areas with > 10% positivity and < 70% of residents vaccinated
*5 (Aug 23, 2021-Mar 11, 2022)*	Aug 23, 2021	Statewide masking order takes effect
	Oct 18, 2021	State deadline for healthcare workers to be vaccinated or have exemption
*6 (Mar 12, 2022-Dec 19, 2022)*	Mar 12, 2022	Statewide masking order rescinded
	Sept 23, 2022	CMS removes recommendation for routine asymptomatic LTCF staff testing
	Oct 31, 2022	State of emergency ended

### Genomic subsampling

Full global data, restricted to those samples with complete date information available, were downloaded from GISAID. Due to the challenges associated with the size of this dataset, we subsampled to include: all sequences from Washington State, 3,000 random sequences from North America, and 3,000 random sequences from regions outside North America to allow for both spatiotemporal diversity and contextualization of LTCF-associated samples in WA. Contextual data included in the phylogenetic analyses were selected from this down-sampled dataset according to genetic proximity to the focal samples (LTCF-associated samples). We specified contextual data sampling to include up to 1,500 genomes per time-period from WA, sampled from all counties and months, ten genomes per month from other US states, and ten genomes per month from each of the global regions. Known duplicate samples were excluded from the contextual sampling.

### Phylogenetic tree generation

Phylogenetic trees corresponding to the six study periods were constructed using Nextstrain SARS-CoV-2 workflow, which aligns sequences against the Wuhan Hu-1 reference using nextalign (https://github.com/nextstrain/nextclade), infers a maximum-likelihood phylogeny using IQ-TREE, and estimates molecular clock branch lengths using TreeTime. We specified the use of discrete trait analysis (DTA) within TreeTime [19, 20].

Data from Yakima LTCFs were separated into two time periods: January-August 2020 and August 2021-December 2022; phylogenetic trees corresponding to each of these time periods were constructed in Nextstrain as described above. These trees were used to select three facilities for further analysis.

### Discrete trait analysis

Migration history was inferred for each of the time-periods using a LTCF-associated binary variable. We defined a migration event into a LTCF as occurring if a parent node had > 50% probability to be assigned the “non-LTCF discrete trait”, and the child node had > 50% probability to be assigned as “LTCF.” The Python library Baltic was used for parsing phylogenetic trees and estimating post-introduction clade sizes (version downloaded from: https://github.com/alliblk/ncov-humboldt/blob/main/baltic.py). [21]. The introduction rate was calculated as the number of unique introduction events over time.

### Genomic epidemiologic analysis

Agreement between clade designation and “outbreak-association” status in the metadata was analyzed for clade sizes > 1. Statewide data were not available for type of association (staff/resident/visitor); age group was evaluated as a proxy to understand possible staff versus visitor introductions. Microreact was used to visualize multiple data elements overlaid on the state-wide phylogenetic trees [22]. Sub-trees for each of the Yakima-specific facilities selected for further analysis were imported into MicrobeTrace for visualization and network analysis [23].

### Transmission tree inference

Time trees from the January-August 2020 analysis for the three Yakima facilities were input into TransPhylo version 1.3.2 to infer transmission trees and describe the role of staff versus resident introduction and transmission events [24, 25]. Previous analyses of SARS-CoV-2 genomic data using TransPhylo were used as reference [26–28]. For this analysis, minimum branch distance was set to one day and viral generation times 1–14 days with a median of 5.5 days and equal sampling time were assumed, [26] along with a gamma distribution. Markov chain Monte Carlo (MCMC) analysis was performed with 500,000 iterations. Convergence was visually inspected.

## Results

**Fig. 1:**
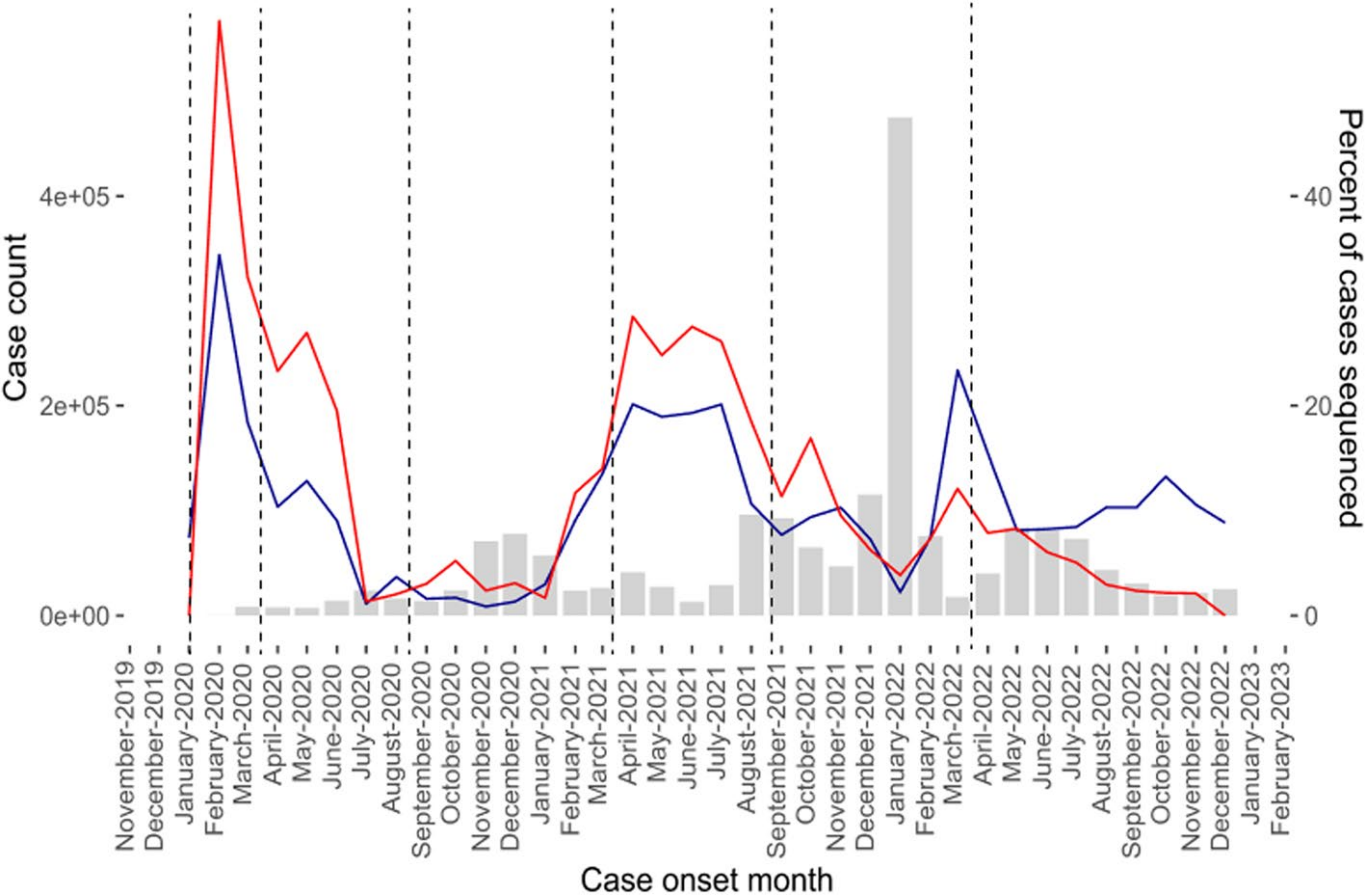
Number of reported cases (gray bars), percent of all cases (blue line) and LTCF-associated cases (red line) sequenced by month, Jan 2020-Dec 2022. The dashed vertical lines indicate the start of each study-time period

Among 58,086 LTCF-associated COVID-19 cases, 4,550 (7.8%) had sequencing performed on at least one specimen. This compares to an average of 9.6% of all reported WA cases with genomic data available. The proportion of cases with sequencing data available varies over time (Fig. 1), ranging from 5 to 30% across study periods. LTCF-associated cases were sequenced at higher frequencies than general-population cases prior to November 2021. During and after November 2021, LTCF-associated cases were sequenced at similar or lower frequency than all cases, with a notable drop-off in sampling beginning in May 2022. A comparison of difference in the percent of LTCF sequencing from the percent of total case sequencing is shown in Supplemental Fig. 1. Sequencing rates vary at the facility- and outbreak-level.

Table 2 compares LTCF-associated cases with sequences available to all LTCF-associated cases. Cases with sequences available were generally demographically representative of all cases by age group, sex, race/ethnicity, language, and facility type but were more likely fatal or hospitalized and were more likely to have symptom information available.

**Table 2:** Comparison of the demographic characteristics between all reported LTCF-associated cases and the subset of those cases with genomic data available (sequenced cases)

	**ALL REPORTED CASES** (***N*** = 58,086)	**SEQUENCED CASES** (***N*** = 4550)
**Sex**		
Female	37,705 (64.9%)	2970 (65.3%)
Male	17,679 (30.4%)	1431 (31.5%)
Other	39 (0.1%)	3 (0.1%)
Missing	2663 (4.6%)	146 (3.2%)
**Age Group**		
0–4	105 (0.2%)	10 (0.2%)
5–17	443 (0.8%)	26 (0.6%)
18–44	15,274 (26.3%)	1062 (23.3%)
45–64	11,177 (19.2%)	836 (18.4%)
65–79	12,174 (21.0%)	1068 (23.5%)
80 +	18,850 (32.5%)	1548 (34.0%)
Unknown	61 (0.1%)	0 (0%)
**Died Due To COVID-19**	4465 (7.7%)	508 (11.2%)
**Hospitalized Due to COVID-19**	7564 (13.0%)	693 (15.2%)
**Outbreak Associated**	37,480 (64.5%)	2781 (61.1%)
**Symptoms**		
Yes	17,014 (29.3%)	1763 (38.7%)
No	7415 (12.8%)	518 (11.4%)
Unknown	33,655 (57.9%)	2269 (49.9%)
**Ethnicity and Race**		
Hispanic	3310 (5.7%)	363 (8.0%)
Non-Hispanic American Indian Or Alaska Native	490 (0.8%)	63 (1.4%)
Non-Hispanic Asian	2265 (3.9%)	191 (4.2%)
Non-Hispanic Black	2494 (4.3%)	166 (3.6%)
Non-Hispanic Multiracial	471 (0.8%)	43 (0.9%)
Non-Hispanic Native Hawaiian Or Other Pacific Islander	372 (0.6%)	33 (0.7%)
Non-Hispanic White	29,429 (50.7%)	2153 (47.3%)
Non-Hispanic Other Race	319 (0.5%)	25 (0.5%)
Unknown	1513 (33.3%)	18,934 (32.6%)
**Language**		
English	13,579 (23.4%)	1256 (27.6%)
Spanish	294 (0.5%)	29 (0.6%)
Other	295 (0.5%)	37 (0.8%)
Unknown	1298 (2.2%)	88 (1.9%)
Missing	42,620 (73.4%)	3140 (69.0%)
**Facility type**		
Adult family home	3764 (6.5%)	282 (6.2%)
Assisted living facility	26,076 (44.9%)	1888 (41.5%)
Facility for individuals with intellectual disability	34 (0.1%)	1 (0.0%)
Nursing home	28,212 (48.6%)	2379 (52.3%)

**Fig. 2:**
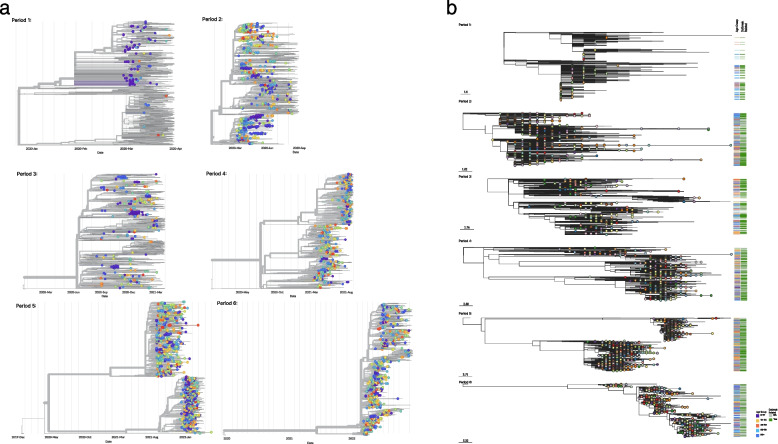
Time-scaled (A) and divergence-scaled (B) Maximum Likelihood phylogenetic trees of sequence data from each study time-period. Divergence-scaled trees include indication of age group and outbreak status for LTCF-associated cases. Nodes are colored by individual facility; colored nodes are LTCF-associated cases, gray nodes are contextual samples

Figure 2 shows time-scaled (A) and divergence-scaled (B) phylogenetic trees of sequenced LTCF cases across all time periods outlined in Table 1. LTCF-associated cases are dispersed and intermixed with both LTCF-associated and non-LTCF cases; across each time-period the dominant lineages match across these groups (Supplemental Fig. 2). Multiple epidemiological clusters within unique facilities are visualized, as well as linked cases from different facilities. Many visualized clusters reveal phylogenetic diversity with long branch lengths, indicating missing samples in the transmission chains consistent with known sampling patterns.

Age-group was evaluated as a proxy for resident status using supplemental data from Yakima County. The oldest age groups, consisting of persons aged 65 and older were > 90% residents. Persons in the 45–64 age group were 43.3% residents; 95.5% of persons 18–44 were staff. Across all time periods, sequences from different age groups are interspersed.

**Fig. 3:**
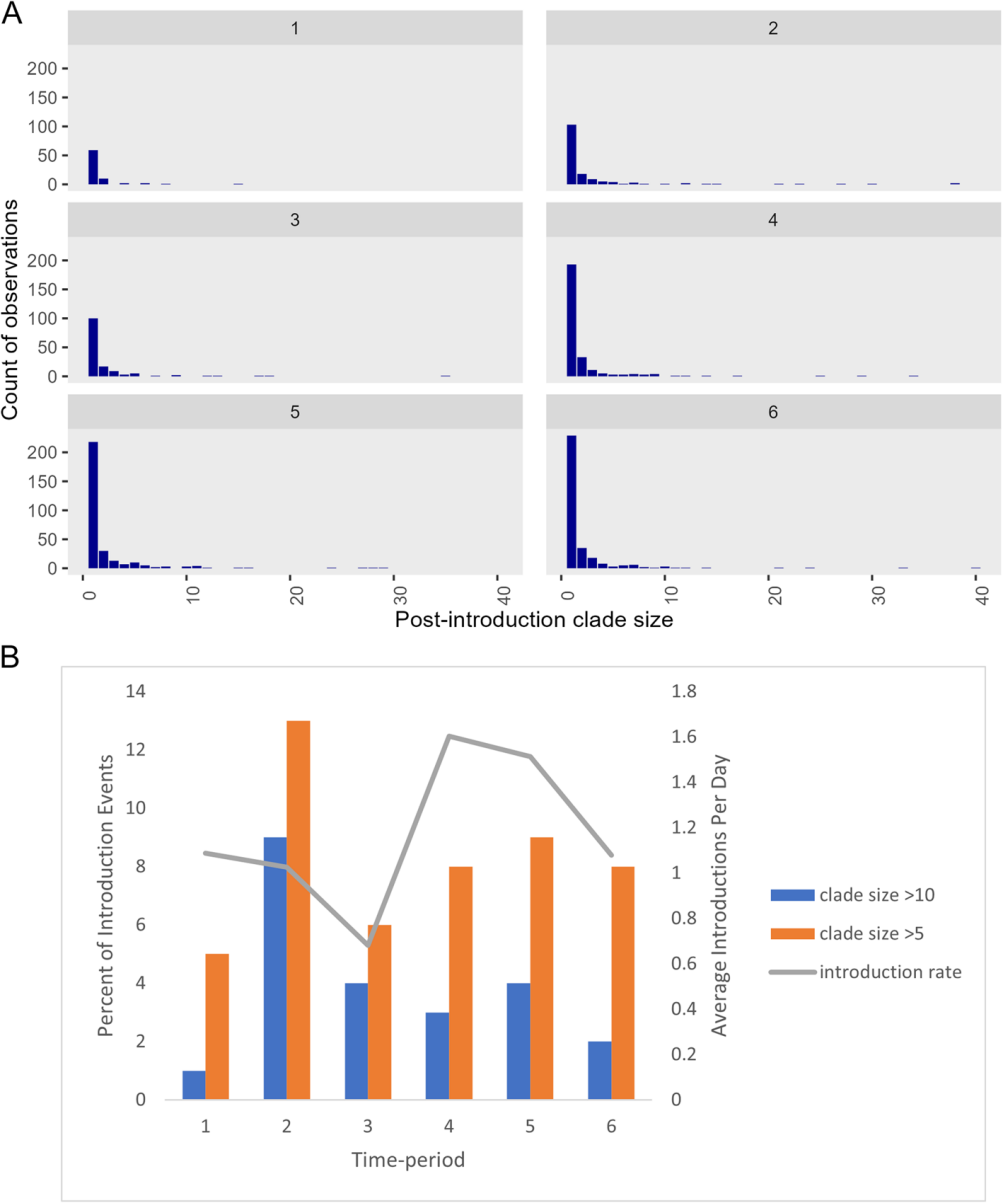
**A** Post-introduction clade sizes among LTCFs in each time period, 1–6*. *Footnote: Additional single observations outside of the figure scale were observed for the following time-periods. Time-period 2: 52, 64, 253, 303. Time-period 3: 51**.** Time-period 5: 57, 405. B Introduction rate (average number of introduction events per day) and percent of introduction events leading to large clade sizes, time-periods 1–6

Figure 3 shows the post-introduction clade sizes among LTCFs in each time-period. Most clusters are single introductions across all time-periods, with large outbreaks (> 10 sequences) becoming increasingly rare. The average number of introductions per day varied from 1.6 during time-period 4 to 0.7 during time-period 3. Additional detail regarding post-introduction clade sizes, introductions per day, and information regarding sampling during each time-period is provided in Supplemental Table 1.

Among cases inferred to be associated with introduction clades sized > 1, varying proportions were labeled as outbreak-associated in the epidemiologic dataset over time, ranging from 49.2%-97.4% (Table 3).

**Table 3:** Agreement of genomic and epidemiologic datasets: proportion of cases marked as outbreak-associated in epidemiologic data among those inferred in LTCF post-introduction clades sized > 1

Time-period	Proportion of cases inferred in LTCF post-introduction clades > 1 and marked as outbreak-associated in epidemiologic datasets
1	56/63 (88.9%)
2	590/1050 (56.2%)
3	262/269 (97.4%)
4	323/382 (84.6%)
5	610/932 (65.5%)
6	223/453 (49.2%)

### Yakima county long-term care facility-associated transmission

Yakima Health District reported supplemental data on 1,725 cases associated with ten facilities; 1,452 (84%) of these case records were linked to WDRS data by probabilistic matching. Genomic data were available for 667 cases. Sequenced cases from Yakima were highly representative based on age, sex, and race. Sequenced cases were more likely to be fatalities (11.1% of sequenced cases vs 8.1% of all facility cases).

**Fig. 4:**
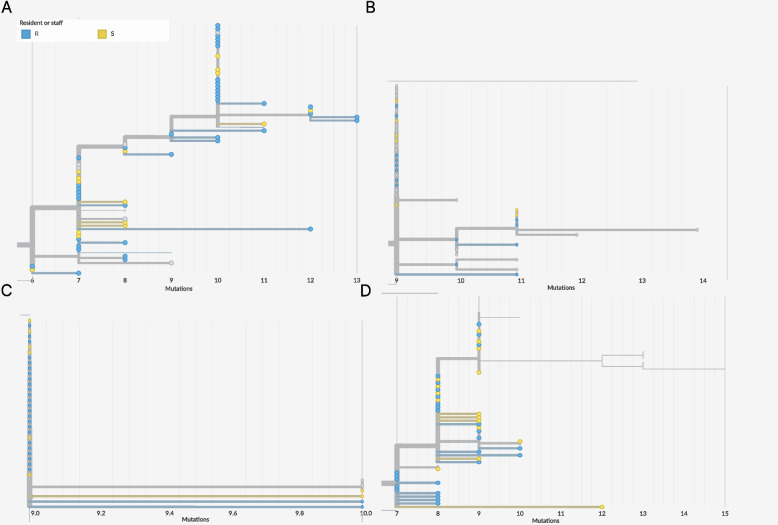
Divergences trees, Yakima County Facilities **A**-**C**. **A** Facility A, April-June 2020. **B**. Facility B-1, March-June 2020. **C**. Facility B-2, March-June 2020. **D**. Facility C, April-Aug 2020

Phylogenetic visualization spanned two time periods, which covered 98% of sequences: January-August 2020 and August 2021-December 2022. Several large facility-associated outbreaks were visualized; three facilities were selected for additional analyses (Supplemental Fig. 3a-b). Facility A was selected due to identification of one prolonged cluster spanning April-June 2020; a divergence tree of each selected outbreak is shown in Fig. 4. Facility B was selected due to two large overlapping outbreaks early in the pandemic with multiple introductions later in the pandemic. Facility C was selected due to apparent multiple introduction events over the course of the pandemic, including early in the pandemic. Resident and staff infections were interspersed across the tree and network visualizations. Trace diagrams resulting from the TransPhylo analysis revealed uncertainty in the parameter values, likely due preponderance of identical consensus genomes, impacting Transphylo’s ability to resolve within- and between-case genetic diversity, as has been described previously for SARS-CoV-2 transmission reconstruction [27].

The Facility A transmission reconstruction inferred 12% of cases as unsampled sources (Supplemental Fig. 4) and inferred a resident as source. During this period, 56% of known cases from Facility A were sequenced (Supplemental Table 2).

An outbreak spanning March 18, 2020 to April 15, 2020 included 27 Facility B sequences; during this period, 58% of known Facility B cases were sequenced. Another 33 sequences from this facility were associated with a separate outbreak spanning April 19, 2020 to May 7 2020.

From April-August 2020, 69% of reported cases from Facility C were sequenced and at least 18 separate introduction events were documented, only one of which apparently led to an outbreak of > 5 cases as visualized in the genomic data. This outbreak included 62 sequences and spanned April 15-May 14, 2020.

The proportion of staff amongst all cases was consistent across these four outbreaks, ranging from 17%-22%. The ratio of observed to expected inferred transmission events attributed to staff ranged from 0.66–1.17, providing evidence that both staff and residents are driving transmission in these outbreaks (Supplemental Table 2).

## Discussion

Here, we analyzed epidemiologic and genomic data associated with LTCFs in WA to characterize transmission dynamics and inform ongoing data utilization. Transmission dynamics in LTCFs changed over the course of the COVID-19 pandemic, with variable introduction rates into LTCFs, but decreasing amplification within LTCFs. Particularly during March-August 2020, a period marked by little population immunity and initiation of non-pharmaceutical interventions, COVID-19 spread in LTCFs via high introduction rates and intra-facility transmission. The number of introduction events and intra-facility clade sizes decreased during August 2020-March 2021; vaccination campaigns began in December 2020. Additionally, CMS released testing requirements for staff and residents in August 2020. Although the introduction rate more than doubled between this time-period and the subsequent two study periods, the percentage of introduction events leading to large clade sizes remained stable. This indicates that despite more frequent introductions during these time periods, post-introduction within-LTCF transmission was curbed, possibly due to vaccination and improved IPC. These study periods were marked by transmission of Delta and Omicron variants, with high levels of community transmission likely contributing to introduction rates. While case counts were high, the genomic data show that incidence was largely driven by repeated introduction events rather than intensive within-LTCF spread.

Over the course of the pandemic, LTCF-associated cases are dispersed throughout the trees and intermixed with both LTCF-associated and non-LTCF cases, indicating that SARS-CoV-2 lineages circulating in LTCFs matched those circulating in surrounding communities. Dominant lineages in each time-period matched when comparing LTCF-associated cases to Washington cases included in the tree. This finding is consistent with a similar study performed in the UK [15]. Similarly, sequences from different age groups are interspersed, indicating likely bi-directional transmission between staff and residents. This observation was validated for a small number of outbreaks, demonstrating proportional inferred transmission from staff and residents.

Interpretation of these findings is limited by variable sequencing over time. For much of the pandemic, testing and sequencing from LTCFs occurred at higher proportions than for the general population of COVID-19 cases. This over-sampling inflates the number of introductions and clade sizes when contextualized among other WA sequences. Changes in the relative proportion of LTCF cases sequenced and in sampling intensity are expected to impact findings of the DTA analysis and comparison across timepoints. However, when considering the direction of expected change, we anticipate the results identified herein are generally a conservative estimate. This conclusion was drawn after comparing the relative direction of change considering sampling proportion and sampling intensity across time-periods to the number of large clades identified. Overall, sequenced LTCF cases were found to be representative of COVID-19 cases in LTCFs.

The potential contribution of genomic data in defining outbreak-related cases was quantified. In the absence of genomic data, outbreak-association is determined using the current Council for State and Territorial Epidemiologists (CSTE) case definition. However, this definition cannot differentiate between concurrent but independent introduction events or outbreaks and relies on epidemiologic data capture. Analysis of the agreement between outbreak-tagged cases in the epidemiological data and cases identified in post-introduction clades sized > 1 revealed that epidemiologic data is growing more disparate from genomic data over time. Specifically, during periods 4–6, cases inferred within LTCF post-introduction clades were less likely to be recorded as outbreak-associated in the epidemiologic datasets compared to during study periods 1–3. This finding suggests that genomic data could greatly inform outbreak definitions, especially in settings of decreased epidemiologic data capture. In the absence of genomic data, outbreaks may also be over-estimated as multiple introduction events are not considered.

Although we attempted transmission reconstruction of four outbreaks in Yakima County, uncertainty in the parameter values limits interpretation of results. Indeed, based on known sequencing rates, TransPhylo estimated fewer missing links than expected and epidemiological data including onset dates provided conflicting results. Methods that utilize additional epidemiological data in reconstruction, such as extension of the outbreaker2 model, may be more useful in this setting [29, 30].

Visualization of this large genomic dataset over time provides insight into useful bioinformatic tools and methods for application in public health practice. Early in the pandemic, many clusters of cases with long persistence were observed. Genomic epidemiology tools often rely on distance thresholds for defining clusters. These tools are difficult to apply in settings of prolonged transmission, as evolution over time is expected. Application of tools requiring thresholds may result in inference of independent clusters in situations of prolonged transmission. This was observed when attempting to use one such tool, MicrobeTrace, in the analysis of outbreaks in Yakima County. In this study, the utilization of DTA analysis with paired epidemiologic data allowed observation of prolonged outbreaks without the need for thresholds.

This study faced several important limitations. First, genomic data captured for LTCF-associated cases were associated with more severe cases. The majority of LTCF-associated outbreaks had no sequences available; this requires an assumption that the sampled LTCFs are representative of the unsampled facilities. Based on our case-level representativeness assessment, including proportional sampling by facility type, we believe this assumption is reasonable. The DTA analysis was performed using a binary variable for LTCF-association; analysis at the facility level may reveal additional introduction events and patterns of inter-facility spread. Demonstrating the relative rarity of large outbreaks caused by a single introduction late in the pandemic is an important finding; however, many guidance, policy, regulation, practice, immunity, and prevention method (including new availability of vaccines) changes occurred over the study period, prohibiting a causal analysis of which component changes led to this impact and limiting our study to observational findings.

This study had several notable strengths. First, we assessed genomic sampling representativeness at the case-level, enabling DTA analysis and interpretation. Second, paired epidemiologic and pathogen genomic data were available with additional detail available for Yakima County cases, facilitating in-depth analysis of transmission. In particular, the ability to de-duplicate sequences early in the pandemic impacted study findings; during the first time-period there were an average of three (triplicative) genomes available among sequenced cases. Analysis in the absence of epidemiologic data will over-represent these cases, inflating genomically-defined clusters. Finally, genomic studies to understand a single or a few outbreaks are commonly performed and reported in the literature. By looking at data over time, we add important context regarding the changing transmission dynamics associated with LTCFs.

Paired genomic and epidemiologic data enable phylogenetic analysis to understand transmission patterns, identify apparent clusters, and form hypotheses regarding transmission networks. However, metadata is not consistently available on some key variables, including type of LTCF association (staff/resident/visitor), dates of association, or travel history. Given currently available data, methods for tree building for hypotheses generation on a routine basis are recommended. Cluster detection tools for outbreak identification are likely of limited use, as most facilities do not have sequencing performed and data is not timely. However, cluster detection on available genomic data may help to identify temporal patterns of intra-facility spread versus repeated introduction. The current data types and quality captured by routine surveillance data collection is inadequate for applying methods to infer transmission or identify introduction sources with certainty. Although this data may be available through enhanced investigations in some counties, as with Yakima County, the general absence of this data limits broader analysis. Importantly, we noted a decrease in data capture from LTCFs over time. Depending on goals for use of genomic data, sentinel surveillance should be increased or targeted surveillance implemented to ensure available data for analysis; likewise, if cluster detection is a desired outcome, data timeliness should be improved.

These findings reflect challenges facing many SARS-CoV-2 genomic data capture systems presently. Antigen-based testing is common but is not compatible with available specimen retrieval practices and sequencing capacity; advances compatible with ongoing genomic data capture are needed. With present patterns of sequencing, LTCFs are underrepresented; expansion to sentinel facilities or during outbreak investigation is recommended. Additionally, genomic epidemiologic workforce capacity embedded within the teams that surveil for outbreaks in healthcare settings is required.

## Conclusions

In conclusion, this analysis identified changing transmission dynamics in LTCFs over the course of the COVID-19 pandemic, with smaller post-introduction clades noted later in the study period despite periods of high introduction rates. This finding is encouraging for the many control efforts that have been put in place in these facilities over time, including vaccination, infection prevention, and testing and reporting to public health jurisdictions, although causal theories could not be tested and natural immunity was also accumulating during this time. LTCFs are likely to remain vulnerable institutions in which ongoing respiratory pathogen monitoring and outbreak control is warranted. Genomic data have the potential to increase the specificity of outbreak detection and resulting public health actions. Ongoing genomic epidemiologic analysis of LTCF-associated data is encouraged to facilitate situational awareness, potential cluster detection, and hypothesis-generation for further targeted analysis.

### Supplementary Information

**Additional file 1: Figure 1. **Difference in percent of LTCF sequencing from percent of total case sequencing by month. **Figure 2. **Proportion of Nextstrain clades among LTCF-associated vs non-LTCF Washington sequences, by time-period. **Table 1. **Percent of introduction events leading to large clades, average introduction events per-day, and sampling proportion and intensity during each time-period. **Figure 3. **Time-scaled phylogenetic tree and divergence scaled phylogenetic tree of sequence data from LTCF-associated cases, Yakima County, January-August 2020 (A), and time-scaled phylogenetic tree from LTCF-associated cases, Yakima County, August 2021-December 2022 (B). Nodes are colored by individual facility; colored nodes are LTCF-associated cases, gray nodes are contextual samples. **Figure 4. **Inferred sampling proportions, Facility A (A), Facility B - Outbreak 1 (B), Facility B - Outbreak 2 (C), Facility C (D). **Table 2. **Sampling and estimated staff contribution to analyzed outbreak, Yakima.

## Data Availability

The data that support the findings of this study are available from Washington State Department of Health (doh.wa.gov) but restrictions apply to the availability of these data, which were used under license for the current study, and so are not publicly available. Data are however available from the authors (Hanna Oltean) upon reasonable request and with permission of Washington State Department of Health.

## Abbreviations

LTCF: Long-term care facility
WA: Washington state
CDC: Centers for Disease Control and Prevention
IPC: Infection prevention and control
PPE: Personal protective equipment
DOH: Washington State Department of Health
CMS: Centers for Medicare and Medicaid Services
WDRS: Washington Disease Reporting System
DTA: Discrete trait analysis
MCMC: Markov chain Monte Carlo
CSTE: Council for State and Territorial Epidemiologists
