# Supplementary material for: Changing genomic epidemiology of COVID-19 in long-term care facilities during the 2020–2022 pandemic, Washington State
Source: BMC Public Health. 2024 Jan 15;24:182. doi: 10.1186/s12889-023-17461-2 (PMC10789038; doi:10.1186/s12889-023-17461-2)
Supplement: Supplementary file 1 — Additional file 1: Figure 1. Difference in percent of LTCF sequencing from percent of total case sequencing by month. Figure 2. Proportion of Nextstrain clades among LTCF-associated vs non-LTCF Washington sequences, by time-period. Table 1. Percent of introduction events leading to large clades, average introduction events per-day, and sampling proportion and intensity during each time-period. Figure 3. Time-scaled phylogenetic tree and divergence scaled phylogenetic tree of sequence data from LTCF-associated cases, Yakima County, January-August 2020 (A), and time-scaled phylogenetic tree from LTCF-associated cases, Yakima County, August 2021-December 2022 (B). Nodes are colored by individual facility; colored nodes are LTCF-associated cases, gray nodes are contextual samples. Figure 4. Inferred sampling proportions, Facility A (A), Facility B - Outbreak 1 (B), Facility B - Outbreak 2 (C), Facility C (D). Table 2. Sampling and estimated staff contribution to analyzed outbreak, Yakima. [file 12889_2023_17461_MOESM1_ESM.docx]

**Supplemental Figure 1:** Difference in percent of LTCF sequencing from percent of total case sequencing by month.


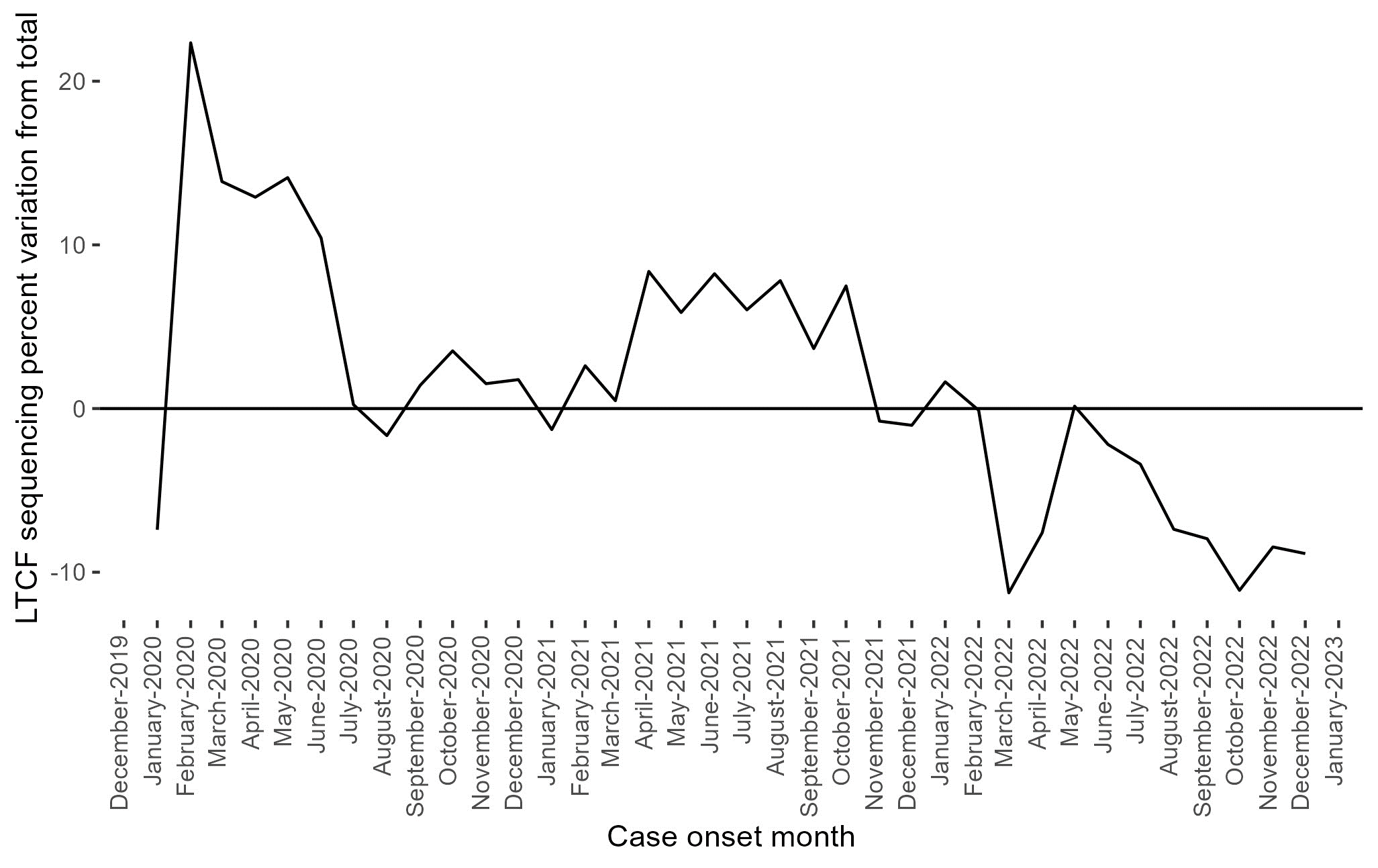


**Supplemental Figure 2:** Proportion of Nextstrain clades among LTCF-associated vs non-LTCF Washington sequences, by time-period.

**Supplemental Table 1:** Percent of introduction events leading to large clades, average introduction events per-day, and sampling proportion and intensity during each time-period.

| Time-period | Percent of introduction events leading to clades >5 | Percent of introduction events leading to clades >10 | Average introductions per day | LTCF sampling deviation from community sampling (percentage points) | Percent of all LTCF cases sequenced |
| --- | --- | --- | --- | --- | --- |
| 1 | 5% | 1% | 1.1 | +9.6 | 30% |
| 2 | 13% | 9% | 1.0 | +8.3 | 18% |
| 3 | 6% | 4% | 0.7 | +1.0 | 5% |
| 4 | 8% | 3% | 1.6 | +6.1 | 23% |
| 5 | 9% | 4% | 1.5 | +0.9 | 11% |
| 6 | 8% | 2% | 1.1 | -6.8 | 5% |

**Supplemental Figure 3:** Time-scaled phylogenetic tree and divergence scaled phylogenetic tree of sequence data from LTCF-associated cases, Yakima County, January-August 2020 (A), and time-scaled phylogenetic tree from LTCF-associated cases, Yakima County, August 2021-December 2022 (C). Nodes are colored by individual facility; colored nodes are LTCF-associated cases, gray nodes are contextual samples.

1. January-August, 2020. Divergence tree displays an alternate view of the figure inset.


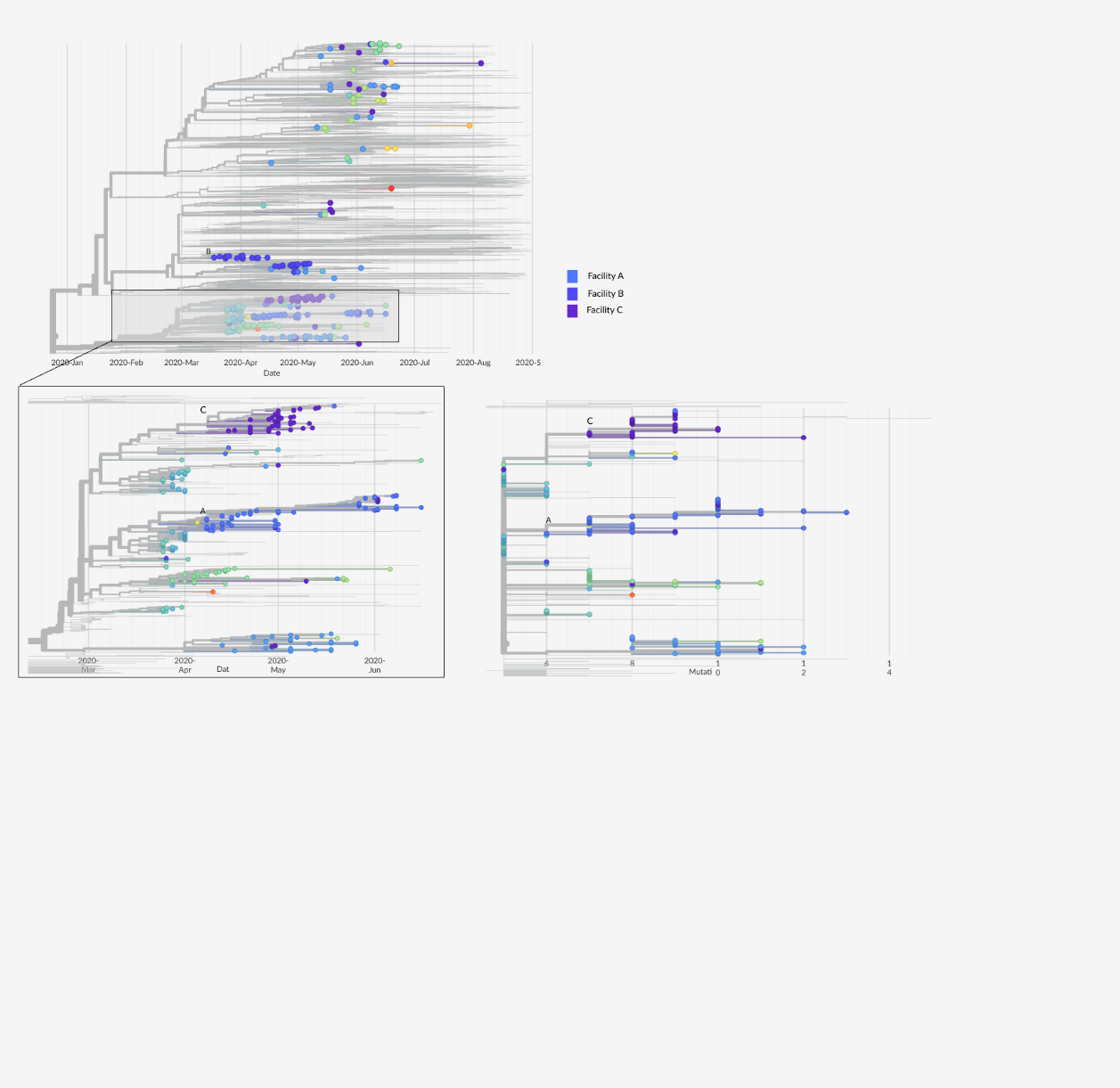


1. August 2021-December 2022


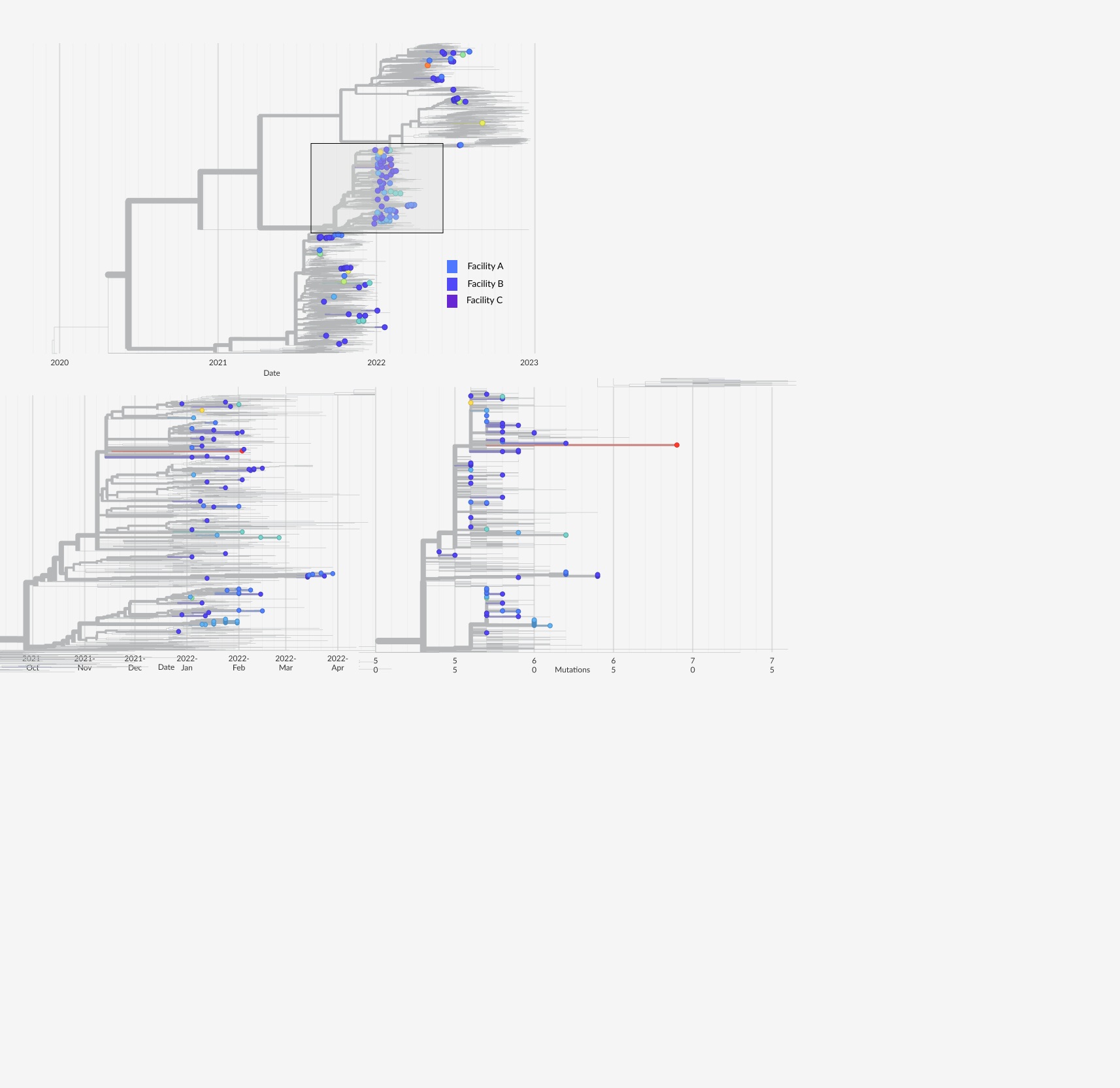


**Supplemental Figure 4:** Inferred sampling proportions

1. Facility A


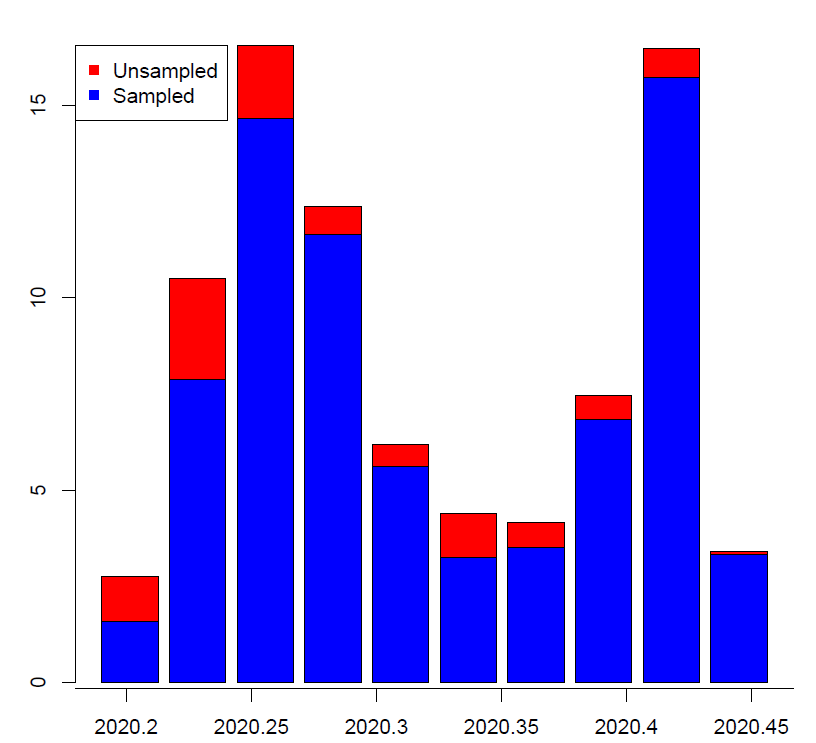


1. Facility B – Outbreak 1


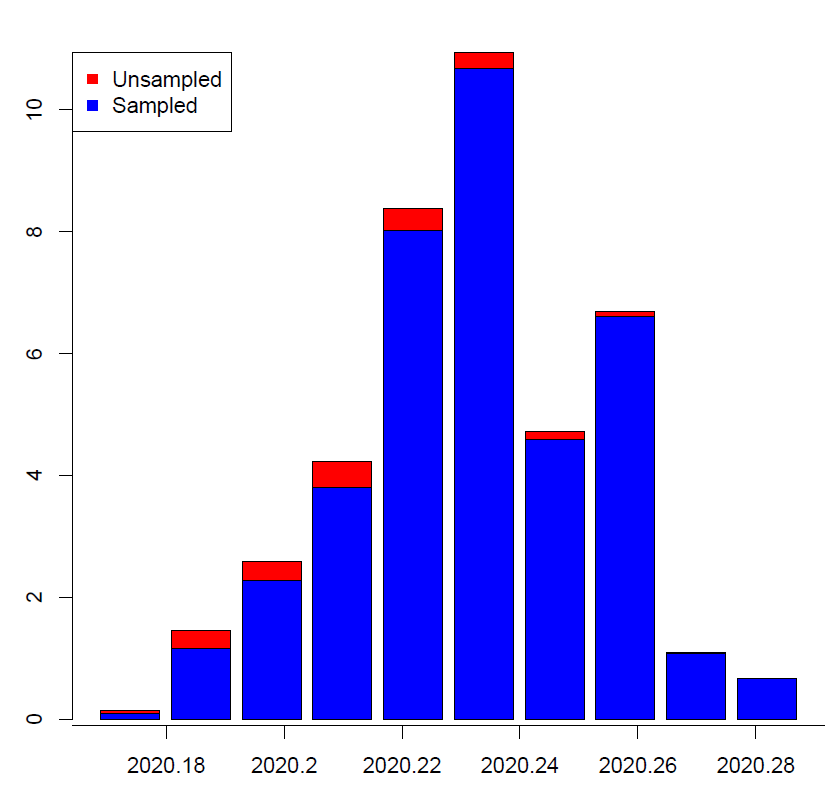


1. Facility B – Outbreak 2


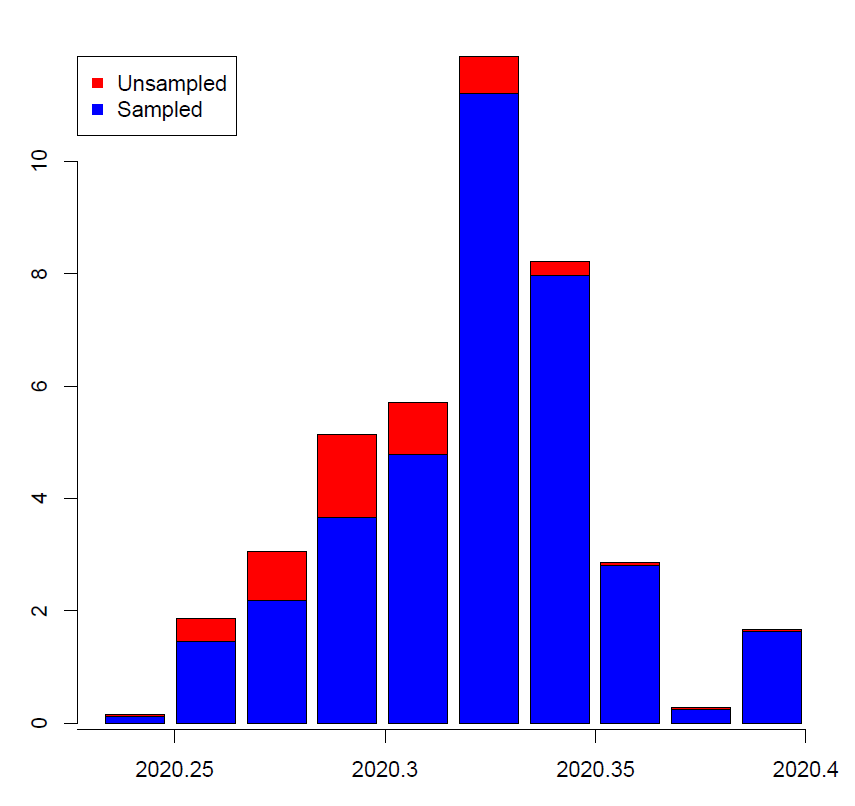


1. Facility C


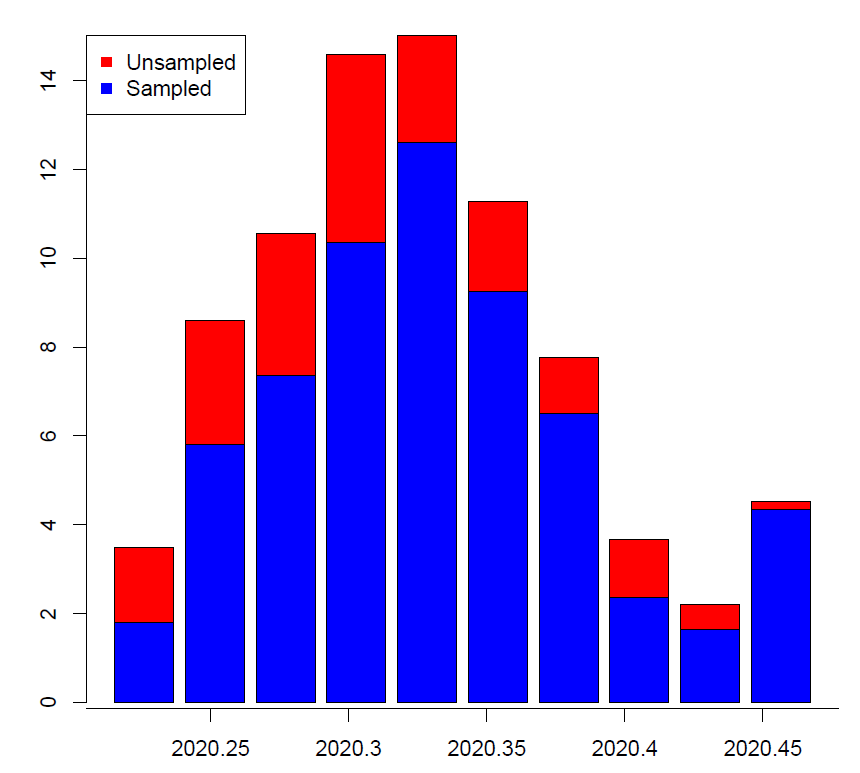


**Supplemental Table 2:** Sampling and estimated staff contribution to analyzed outbreak, Yakima

| Facility/Outbreak | Sequencing proportion during period of interest | Transphylo inferred sampling | Proportion of staff among cases | Observed/Expected transmission events attributed to staff |
| --- | --- | --- | --- | --- |
| A | 56% | 88% | 0.18 | 0.94 |
| B-1 | 58% | 95% | 0.17 | 1.17 |
| B-2 | 58% | 95% | 0.21 | 0.90 |
| C | 69% | 79% | 0.22 | 0.66 |
